# Supplementary material for: Large language models for psychosocial risk assessment: A multi-method evaluation across suicide, intimate partner violence, and substance misuse
Source: PLOS Digit Health. 2026 Apr 27;5(4):e0001352. doi: 10.1371/journal.pdig.0001352 (PMC13120283; doi:10.1371/journal.pdig.0001352)
Supplement: S1 Table — Values represent means (standard deviations) for Likert-scale ratings assessing perceived accuracy of the vignette, quality, relevance, and empathy of responses, as well as the quality and relevance of risk assessment questions and treatment recommendations. Percentages indicate the proportion of participants who agreed with suggested actions, reported consistency with past actions, and felt understood by the model. (DOCX) [file pdig.0001352.s001.docx]

**Table S1**

The Full Results for Study 2

|  | **Suicide** | | | **IPV** | | | **Substance misuse** | | | **Overall** | | |
| --- | --- | --- | --- | --- | --- | --- | --- | --- | --- | --- | --- | --- |
|  | **GPT**  **(*n* = 20)** | **Claude**  **(*n* = 16)** | **Total**  **(*n* = 36)** | **GPT**  **(*n* = 22)** | **Claude**  **(*n* = 17)** | **Total**  **(*n* = 39)** | **GPT**  **(*n* = 22)** | **Claude**  **(*n* = 13)** | **Total**  **(*n* = 35)** | **GPT**  **(*n* = 64)** | **Claude**  **(*n* = 46)** | **Total**  **(*n* = 111)** |
| *Accuracy of the vignette^a^* | *3.35 (1.27)* | *2.88 (1.15)* | *3.14 (1.22)* | *3.36 (1.26)* | *3.53 (0.94)* | *3.44 (1.12)* | *3.14 (1.08)* | *2.62 (1.12)* | *2.94 (1.11)* | *3.28 (1.19)* | *3.04 (1.11)* | ***3.16 (1.17)*** |
| Quality of response | 4.00 (0.97) | 3.44 (1.03) | 3.75 (1.02) | 3.91 (0.81) | 3.41 (0.87) | 3.69 (0.86) | 3.32 (1.21) | 4.31 (1.11) | 3.69 (1.25) | 3.73 (1.04) | 3.67 (1.06) | **3.70 (1.04)** |
| Relevance of response | 4.05 (1.05) | 3.81 (0.91) | 3.94 (0.98) | 4.00 (0.87) | 3.71 (0.77) | 3.87 (0.83) | 3.68 (1.21) | 4.54 (0.66) | 4.00 (1.11) | 3.91 (1.05) | 3.98 (0.86) | **3.94 (0.97)** |
| Empathy of response | 4.10 (1.07) | 3.50 (1.03) | 3.83 (1.08) | 3.95 (0.90) | 4.24 (0.83) | 4.08 (0.87) | 3.30 (1.34) | 4.31 (0.75) | 3.70 (1.24) | 3.79 (1.15) | 4.00 (0.94) | **3.85 (1.10)** |
| Quality of risk questions | 4.25 (0.72) | 3.81 (0.91) | 4.06 (0.83) | 4.14 (0.83) | 4.24 (0.75) | 4.18 (0.79) | 3.45 (1.10) | 4.23 (0.93) | 3.76 (1.09) | 3.95 (0.95) | 4.09 (0.86) | **4.02 (0.91)** |
| Relevance of risk questions | 4.45 (0.76) | 4.06 (0.77) | 4.28 (0.78) | 4.27 (0.70) | 4.24 (0.83) | 4.26 (0.75) | 3.55 (1.10) | 4.54 (0.66) | 3.94 (1.06) | 4.10 (0.94) | 4.26 (0.77) | **4.17 (0.87)** |
| Quality of treatment options | 4.30 (0.80) | 3.69 (1.01) | 4.03 (0.94) | 4.14 (0.71) | 4.18 (0.73) | 4.15 (0.71) | 3.58 (1.17) | 4.00 (1.08) | 3.75 (1.14) | 4.02 (0.94) | 3.96 (0.94) | **3.98 (0.94)** |
| Relevance of treatment options | 4.35 (0.88) | 4.00 (1.03) | 4.19 (0.95 | 4.14 (0.77) | 4.35 (0.70) | 4.23 (0.74) | 3.58 (1.22) | 4.38 (0.87) | 3.91 (1.15) | 4.03 (1.00) | 4.24 (0.87) | **4.11 (0.95)** |
| % Agree with actions suggested | 85.0% | 87.5% | 86.1% | 90.9% | 88.2% | 89.7% | 77.3% | 92.3% | 82.9% | 84.4% | 89.1% | **86.5%** |
| % Consistent with past actions* | 92.9% | 90.9% | 92.0% | 87.5% | 90.0% | 88.5% | 72.7% | 72.7% | 78.3% | 88.1% | 84.4% | **86.5%** |
| % Felt understood | 70.0% | 56.3% | 63.9% | 68.2% | 82.4% | 74.4% | 65.0% | 92.3% | 75.8% | 65.6% | 76.1% | **70.6%** |
