# Supplementary material for: Large language models for psychosocial risk assessment: A multi-method evaluation across suicide, intimate partner violence, and substance misuse
Source: PLOS Digit Health. 2026 Apr 27;5(4):e0001352. doi: 10.1371/journal.pdig.0001352 (PMC13120283; doi:10.1371/journal.pdig.0001352)
Supplement: Attachment — Submitted filename: Comments .docx [file pdig.0001352.s002.docx]

This work evaluates whether large language models (LLMs) can support scalable psychosocial risk assessment across suicide, intimate partner violence, and substance misuse. Across the three studies, GPT-4, Claude, and a supervised three agent GPT-4o system showed strong accuracy in detecting psychosocial risk and aligning with participant rated severity despite challenges with suicidality while participants rated LLM responses as accurate, empathic, and clinically useful, and the chatbot reliably completed structured assessments using a JSON-based protocol. Overall, the findings show that LLMs can meaningfully aid psychosocial risk evaluation while revealing both their potential and their current limitations in mental health care.

These are several comments for the manscipt below:

1. Please change all possessive case such as participants’ lived experiences, participants’ gender identity, researchers’ analysisand participants’ perceptions.They may look like AI writing shallow rewrite by the authors.
2. The authors used Amanda to build the agents. It is not a popular and power tool to build LLM agents. Therefore, I recommend that the authors instead implement the framework in **Python**, which is standard and more robust for constructing LLM agents.

This manuscript presents research focused more on psychosocial issues and related solutions than on medical health, and I recommend transferring it to **PLOS Mental Health.**
