# Supplementary material for: Large language models for psychosocial risk assessment: A multi-method evaluation across suicide, intimate partner violence, and substance misuse
Source: PLOS Digit Health. 2026 Apr 27;5(4):e0001352. doi: 10.1371/journal.pdig.0001352 (PMC13120283; doi:10.1371/journal.pdig.0001352)
Supplement: Attachment — Submitted filename: 260114PLOS_revision.docx [file pdig.0001352.s004.docx]

We thank the editor and the reviewers for giving us an opportunity to revise and submit our paper titled, “Large Language Models for Psychosocial Risk Assessment: A Multi-Method Evaluation Across Suicide, Intimate Partner Violence, and Substance Misuse”, for consideration in PLOS Digital Health. We would like to thank the editor and reviewers for their thorough and extremely helpful feedback in improving our manuscript. We have responded to all reviewers’ comments after each comment in the response letter below and copied and pasted the changes made to the manuscript after each comment where relevant.

**Additional Editor Comments (if provided):**

**Thank you for submitting your manuscript to our journal. We have now received feedback from two expert reviewers, whose comments are appended below. Both reviewers acknowledge the importance of your work, particularly its multi-method design, ecological validity through lived-experience vignettes, and innovative multi-agent architecture for safe deployment. The reviewers have also identified several areas where the manuscript can be strengthened. Given the constructive nature of these comments and the overall potential of your study, the editorial decision is Major Revision. We invite you to address all points raised by the reviewers in a revised manuscript. Please submit a detailed point-by-point response explaining how each comment has been addressed. We believe these revisions will significantly enhance the clarity, rigor, and impact of your valuable contribution to the field of digital health and AI-assisted psychosocial assessment. We look forward to receiving your revised manuscript.**

>>> Thank you. We very much appreciate the opportunity to revise the manuscript.

Reviewer 1:

**General Comments
This study explores the efficacy of large language models (LLMs) for psychosocial risk assessment across three high-stakes risks: suicidality/self-harm, intimate partner violence (IPV) and substance misuse. It expands the scope of multi-domain assessments which are used frequently in the existing literature but generally includes suicide only. The research proposes a supervised multi-agent composition to apply a role-specialized architecture providing collaborative technologies including a therapeutic agent, supervisor, and structured assessor and applying JSON schema to generate structured output about risk assessment. The use of vignettes that derive from lived-experience data, rather than entirely synthetic prompts, is a good step towards enhancing ecological validity. While these are impressive strengths of the manuscript, there are also several areas that are weak and require a revision.**
>>> We appreciate the acknowledgement of the strengths of the present study and the thoughtful comments to help improve the manuscript.

Specific Comments
**Introduction
According to Clay (2021), it is estimated that mental health apps number from 10,000 to 20,000. This data seems old. There have been large-scale launches of applications based on LLMs since 2022. Therefore, updating this with recent data may be a good idea.**
>>> We have updated the reference to 2025 which suggests that there are more than 20,000 apps.

**Calling ELIZA an “AI” is technically not correct because it is just a Rule-based System. Please adjust this terminology for precision.**
>>> The terminology ‘AI’ has traditionally been used precisely to describe rule-based computer systems before predictive modelling and machine learning was possible. In the paper, we describe what first generation AI was like in the sentence mentioning Eliza. Specifically, we state: *“The invention of Eliza in 1966 marked the beginning of conversational AI, but for decades chatbot development was constrained by decision tree and rule-based approaches, which relied on predefined scripts and conditional ‘if–then’ rules to generate responses rather than genuine language understanding”.* We believe this is factually correct and have not changed the wording.

**Study 1
Reproducibility: The specific version of the Claude model used must be disclosed. To ensure reproducibility, please provide full prompt texts, model versions/dates, temperature and decoding settings, and any system/role prompts used across the studies.**

>>> We have added the specification in the methods: “Two LLMs were tested: GPT-4 (OpenAI) and Claude 3 Sonnet (Anthropic). Both models were accessed programmatically via their respective APIs—OpenAI’s GPT-4 (version 20240613) via openai.ChatCompletion.create() and Claude 3 Sonnet (version 20240229) via the Anthropic API using the anthropic.HUMAN_PROMPT and anthropic.AI_PROMPT format.” We have also made an example python script available on the Open Science Framework project page. All scripts for the different risks and models were similar in terms of temperature, prompts etc. so this should provide all the information necessary.

**What standardize instruments or expert adjudication (if any) were use to validate the severity labels? If none were used, please explain why participant self-rating was chosen rather than clinician ratings or established validated scales.**

>>> We have added an explanation in the current study section: *“Furthermore, participants’ own ratings of the highest level of risk they believed they had experienced were used as the primary benchmark for severity classification. Although clinician-administered tools are widely used in practice, decades of research demonstrate that even structured professional judgment has only modest predictive accuracy for suicide and other forms of psychosocial risk (Franklin et al., 2017; Woodford et al., 2019). Moreover, the aim of the present study was not to predict future outcomes but to examine whether LLMs could interpret lived-experience narratives in ways that align with individuals’ perceived severity at the time. Participant self-ratings therefore provided a theoretically consistent reference point for evaluating concordance between human and model interpretations, while acknowledging that such ratings are not equivalent to formal clinical diagnosis or actuarial prediction.”*

**Bias Control: During the development of the vignettes, each case has been drafted by researchers according to the data provided by participants. Those researchers who used these stories are biased. How was the potential inter-researcher bias dealt with by the team?**

>>> We are unsure what the reviewer refers to here. We only used information provided by the participants to develop the vignettes which were largely developed by providing ChatGPT with the participants’ responses based on several researcher-written examples and checking the vignettes for accuracy. No other details were added to the vignettes so there is very little possibility of a bias. We have added this for further clarity: *“No additional information beyond what the participants had provided in the survey were included in the vignettes.”*

**Study 2
The OpenAI API and the Anthropic API are used in the study. It does not test any local models. Local models may perform worse but are better for patient privacy. There is a need to test whether the results hold using local models to evaluate the privacy-generalization trade-off.**

>>> We have added this in future directions: *“Fifth, all models evaluated in the present research were accessed via commercial APIs (OpenAI and Anthropic), and we did not test locally hosted or open-weight models. While API-based systems currently offer state-of-the-art performance, locally deployable models may differ in capability and raise distinct privacy advantages. Future work should therefore examine whether comparable results hold using on-premise or open-source models to better evaluate the privacy–performance trade-off in clinical contexts.”*

**The presently available information is barely adequate for comparing the two models. I recommend the use of certain statistical methods, like Semantic Textual Similarity, to more firmly prove that the outputs of the models are not different.
Another evaluation metric or benchmark would ensure it is easier to interpret and compare Study 2.**

>>> The primary purpose of Study 2 was not to compare model performance but to more generally evaluate how well LLMs can evaluate risk and how participants felt about the evaluations. Therefore, we have not included any further analysis at this stage although this would be interesting in future work which aims to compare the efficacy of different models.

**Study 3
Forcing tokens to output strict JSON formats can cause failures in LLM handling. How did the researchers deal with “bad JSON” or parsing errors?**

>>> As can be seen in Table 4, we report whether each of the questions was asked by the LLMs. We did not have any ‘bad JSON’ or parsing errors.

**The system's completion of the assessments "Most of the time" is not sufficiently specific. The manuscript on research project lacks numbers. Such as completion rate, detection latency, assessment coverage and safety violation rate.**
>>> Please note that the numbers are presented in Table 4. The assessments were always completed because the research assistants were instructed to go through the procedure until the end. We state this clearly in the results: *“Across all three domains—suicide/self-harm, IPV, and substance misuse—the chatbot successfully completed 100% of the interactions, as defined by our criteria for conversational completion: the dialogue ended with a clear closing phrase (e.g., "thank you", "goodbye"), and a solution or summary response was provided.”*

General Discussion
The relation work section needs to discuss more in depth previously conducted standardized, structured suicide assessments (with LLMs), psychiatry-specific benchmarks and human evaluation (e.g. C-SSRS mapping, PsychiatryBench, QUEST)

>>> We have added this in the introduction already as we felt it would be important to introduce these benchmarks already in the introduction: *“Recent work has begun to formalize evaluation of LLMs in psychiatry using standardized benchmarks. For example, efforts to map LLM outputs onto structured suicide assessment frameworks such as the Columbia–Suicide Severity Rating Scale (C-SSRS; Salvi, 2019) have examined whether models can classify ideation intensity, intent, and planning dimensions in ways consistent with clinical coding. Psychiatry-specific benchmarks such as PsychBench (Liu et al., 2025) and QUEST (Tam et al., 2024) have further evaluated LLM reasoning across psychiatric diagnostic and risk-related scenarios using structured scoring rubrics and human expert adjudication. More recently, dedicated mental health LLM benchmarks including MindBench (Dwyer et al., 2025) and VeraMH (Bentley et al., 2026) have been introduced to assess safety, clinical reasoning, and response appropriateness across sensitive domains. These developments signal a shift from informal vignette testing toward standardized, reproducible evaluation frameworks. The present study complements these approaches by focusing on multi-domain psychosocial risk and by integrating structured JSON-based assessment within a live conversational architecture rather than evaluating isolated prompt–response pairs.”* Note that some of these benchmarks (MindBench and VeraMH) came out after our paper was written but we have included them for completeness.

We have also added this in the discussion:

*“These findings should also be interpreted in light of the growing movement toward standardized mental health benchmarks for LLM evaluation (e.g., PsychBench, QUEST, MindBench, VeraMH). Unlike these benchmarks, which primarily assess model performance on fixed prompt–response tasks or expert-scored scenarios, the present work examined multi-domain psychosocial risk using lived-experience–derived cases and extended evaluation into supervised conversational deployment. As such, our results speak to feasibility under structured, staged conditions rather than to benchmark superiority or comprehensive clinical validation.”*

**The study does not include a clinician baseline and does not compare to any validated screening tool. It fails to match against traditional Machine Learning baselines or fine-tuned encoder/decoder models. The least that the discussion should do is show or argue that LLMs do better than machine learning on these tasks.**
>>> The purpose of the study was not to compare LLMs to other approaches but to simply understand whether LLMs could detect risk and conduct a risk assessment, and how participants felt about this. We have added this in the limitations: *“Furthermore, the present studies did not include a clinician comparison group, validated screening tool benchmark, or traditional machine learning baseline, and therefore do not allow direct conclusions about whether LLMs outperform human assessors or alternative modeling approaches in psychosocial risk evaluation.”*

**Reviewer #2: This work evaluates whether large language models (LLMs) can support scalable psychosocial risk assessment across suicide, intimate partner violence, and substance misuse. Across the three studies, GPT-4, Claude, and a supervised three-agent GPT-4o system showed strong accuracy in detecting psychosocial risk and aligning with participant-rated severity despite challenges with suicidality, while participants rated LLM responses as accurate, empathic, and clinically useful, and the chatbot reliably completed structured assessments using a JSON-based protocol. Overall, the findings show that LLMs can meaningfully aid psychosocial risk evaluation while revealing both their potential and their current limitations in mental health care.**

>>> We would like to thank the reviewer for taking the time to review the manuscript.

**These are several comments for the manscipt below:
1. Please change all possessive case such as participants’ lived experiences, participants’ gender identity, researchers’ analysis and participants’ perceptions. They may look like AI writing shallow rewrite by the authors.**
>>> We are not quite sure why these would need to be changed as this is correct in English and how we would ourselves write. Therefore, we have not changed the language although if the editor feels like this should be changed, we can do so.

**2. The authors used Amanda to build the agents. It is not a popular and powerul tool to build LLM agents. Therefore, I recommend that the authors instead implement the framework in Python, which is standard and more robust for constructing LLM agents.**
>>> There seems to be a misunderstanding. Amanda is a chatbot which we have previously developed based on GPT-4o LLM. The development itself was done in Python.

**Reviewer 3:

This manuscript addresses a highly topical and important question: whether large language models can meaningfully contribute to psychosocial risk assessment across suicidality, intimate partner violence, and substance misuse. Overall, the paper is ambitious, carefully executed, and clearly written, and it goes well beyond much of the existing work in this area, which tends to rely on simplified prompts or single-domain evaluations. The multi-method design, in particular, is a real strength, and the authors should be commended for attempting to bridge technical performance, user perceptions, and system-level implementation within a single programme of research. One of the most valuable aspects of the paper is its grounding in lived experience. Constructing vignettes from participants’ own disclosures, rather than from hypothetical or researcher-generated cases, substantially improves ecological validity and allows for a more realistic assessment of what current LLMs can and cannot do. Likewise, the decision to include IPV and substance misuse alongside suicidality is welcome, as it reflects the complexity of real clinical practice and avoids the narrow focus that characterises much of the existing literature.**

>>> We very much appreciate the acknowledgement of the strengths of the work.

**That said, there are several areas where the manuscript would benefit from a more cautious and critical treatment of its findings. In Study 1, the reported 100% accuracy for domain detection by both GPT-4 and Claude is striking, but also raises questions about how challenging the task actually was. Given the way the vignettes were constructed and prompted, it seems likely that domain identification was relatively explicit. This does not undermine the study, but it does mean that these results should be interpreted carefully and framed as a baseline rather than as evidence of robust real-world performance. Relatedly, the use of participants’ own ratings of risk severity as the comparison standard is understandable given the study aims, but it is not equivalent to a clinical benchmark. The paper would be stronger if this limitation were acknowledged more explicitly, and if the implications of relying on subjective self-assessment were discussed in greater depth.**

>>> We have added these in the Study 1 discussion:

*“The 100% domain identification observed for both models should be interpreted as performance under structured and single-domain conditions. Because vignettes were constructed to reflect one primary risk type and did not include comorbid or deliberately ambiguous presentations, these findings represent a baseline test of categorical detection rather than a proxy for real-world diagnostic complexity. Accordingly, these findings should not be taken as evidence of robust real-world performance in uncontrolled clinical environments, where disclosures are fragmented, multi-domain, and contextually embedded.”*

We also our decision to use participant-ratings in the current study section now based on Reviewer 1’s comment: *“Furthermore, participants’ own ratings of the highest level of risk they believed they had experienced were used as the primary benchmark for severity classification. Although clinician-administered tools are widely used in practice, decades of research demonstrate that even structured professional judgment has only modest predictive accuracy for suicide and other forms of psychosocial risk (Franklin et al., 2017; Woodford et al., 2019). Moreover, the aim of the present study was not to predict future outcomes but to examine whether LLMs could interpret lived-experience narratives in ways that align with individuals’ perceived severity at the time. Participant self-ratings therefore provided a theoretically consistent reference point for evaluating concordance between human and model interpretations, while acknowledging that such ratings are not equivalent to formal clinical diagnosis or actuarial prediction.”*

We also include this in the limitations: *“Furthermore, the present studies did not include a clinician comparison group, validated screening tool benchmark, or traditional machine learning baseline, and therefore do not allow direct conclusions about whether LLMs outperform human assessors or alternative modeling approaches in psychosocial risk evaluation.”*

**Study 2 is, in my view, one of the strongest parts of the manuscript. Bringing participants back to evaluate the AI-generated responses adds an important human perspective that is often missing from evaluations of mental health chatbots. The generally positive ratings for empathy, relevance, and usefulness are encouraging. However, I think the authors underplay one important finding here: around 30% of participants did not feel understood by the chatbot. In contexts involving psychosocial risk, this is not a trivial minority. Several participants reportedly described the responses as generic or “robotic”, or noted that a chatbot could not truly understand their situation. These comments deserve more analytical attention, as they point to the limits of perceived empathy and the potential consequences for trust and engagement.**

>>> We appreciate the reviewer’s acknowledgement of the strength of Study 2 and having participants evaluate the LLM responses. We have amended the discussion in Study 2 which now states: *“At the same time, the finding that nearly 30% of participants did not feel understood warrants careful consideration. In the context of psychosocial risk, this represents a clinically meaningful minority. Qualitative feedback describing responses as generic or “robotic” suggests that the presence of empathic language does not automatically translate into perceived relational attunement. While LLMs may accurately detect risk and generate supportive phrasing, perceived understanding appears to depend not only on linguistic markers of empathy but also on users’ sense of authenticity and contextual depth in the interaction. In high-risk settings, such perceptions may influence trust, disclosure, and engagement with recommended actions. These findings therefore indicate that technical accuracy and empathic wording are necessary but not sufficient for clinical acceptability.”*

**Study 3 is clearly the most innovative component of the paper and represents a meaningful step forward in thinking about how LLMs might be deployed safely. The multi-agent architecture, with a supervisory model and a structured JSON-based assessor, is well motivated and thoughtfully designed. The findings suggest that such a system can, in most cases, complete structured risk assessments while maintaining a reasonably therapeutic tone. At the same time, the results also highlight important failure modes, particularly when the system assumes that certain questions have already been answered or shifts prematurely to supportive action at the expense of assessment completeness. These moments are arguably where the most clinically relevant insights lie, and I would encourage the authors to engage with them more critically rather than treating them primarily as minor implementation issues.**

>>> We have added this in the Study 3 discussion: *“These failure cases are not merely minor implementation issues but illustrate a structural tension between conversational responsiveness and protocol fidelity. In prioritizing therapeutic flow or inferring that a question had already been answered, the system at times privileged relational coherence over assessment completeness. In safety-critical contexts, such assumptions could result in incomplete data capture if mandatory risk indicators are not explicitly confirmed. Future iterations should therefore incorporate explicit completeness checks or hard constraints to ensure that all required assessment items are addressed before transitioning to supportive intervention.”*

**Across the paper, the discussion sometimes comes close to overstating what the findings can support. The models show good agreement with participants and are perceived as helpful, but this should not be conflated with clinical adequacy or safety in real-world deployment. A clearer distinction between risk detection, risk formulation, and risk prediction would help here, as would a more explicit statement about what these systems should not be used for. The ethical discussion is thoughtful, but it could be strengthened by more direct engagement with issues of responsibility, false reassurance, and the potential consequences of missed or underestimated risk.**

>>> We have addressed this throughout the discussion. For example, we have added this in the beginning of the general discussion: “*The present studies primarily evaluate detection and structured assessment alignment under controlled conditions and do not test prospective predictive validity or real-world clinical safety.”* In the implications, we say: *“For practice, the findings suggest that LLMs may assist with structured information gathering and preliminary triage under supervision, but they should not be used as standalone decision-makers for determining clinical risk level, discharge safety, or emergency intervention.”* And *“A central ethical concern is also the possibility of false reassurance. If a system underestimates severity or prematurely transitions to supportive dialogue without completing assessment, users may interpret this as confirmation that their risk is low. In safety-critical contexts, such miscalibration could delay help-seeking or reduce disclosure. Responsibility for oversight therefore cannot be delegated to the model itself; accountability must remain with clinicians, institutions, and developers who define deployment boundaries and monitoring mechanisms.”*

We have also amended the conclusion in the abstract: *“Together, these studies suggest that LLMs can contribute to psychosocial risk detection and structured assessment under controlled conditions, while underscoring the need for careful supervision, rigorous validation, and clearly defined boundaries before consideration of real-world clinical deployment.”*

**From a presentation standpoint, the manuscript is generally well organised and readable. Some sections of the introduction are longer than necessary and could be tightened, but this is a relatively minor issue. The open science practices are a clear strength, and the availability of materials and data will be valuable for the field.**

>>> We appreciate the positive comments. We have not tightened the introduction but if there are specific aspects of the introduction that appear too wordy, we’re open to revising it.

**In summary, this is a strong and important paper that makes a genuine contribution to research on LLMs in mental health and psychosocial risk assessment. However, the interpretation of the results would benefit from greater restraint, and the limitations—particularly around benchmarks, task difficulty, and failure cases—need to be more fully acknowledged and integrated into the discussion. For these reasons, I would recommend major revisions. With these revisions, the manuscript has the potential to become a highly influential reference in this rapidly developing area.**

>>> We very much appreciate the constructive comments by the reviewer and we hope that the revised version acknowledges the limitations more fully and that we have addressed the reviewer’s comments.

**Please see below the journal requirements:**

**1. Please amend your detailed Financial Disclosure statement. This is published with the article. It must therefore be completed in full sentences and contain the exact wording you wish to be published.**

**i. Please clarify all sources of financial support for your study. List the grants, grant numbers, and organizations that funded your study, including funding received from your institution. Please note that suppliers of material support, including research materials, should be recognized in the Acknowledgements section rather than in the Financial Disclosure.**

**ii. State the initials, alongside each funding source, of each author to receive each grant. For example: "This work was supported by the National Institutes of Health (####### to AM; ###### to CJ) and the National Science Foundation (###### to AM)."**

**iii. State what role the funders took in the study. If the funders had no role in your study, please state: “The funders had no role in study design, data collection and analysis, decision to publish, or preparation of the manuscript.”**

**iv. If any authors received a salary from any of your funders, please state which authors and which funders.**

>>>> We only received institutional funding and have amended the financial statement in line with the journal requirements: *“The research was funded by the Institute of Psychology at University of Lausanne, Switzerland (to LMV). The funders had no role in study design, data collection and analysis, decision to publish, or preparation of the manuscript.”*

**2. Please ensure that your Ethics Statement is available in its entirety at the beginning of your Methods section, under a subheading 'Ethics Statement'. It must include:**

**i) The name(s) of the Institutional Review Board(s) or Ethics Committee(s)**

**ii) The approval number(s), or a statement that approval was granted by the named board(s)**

**iii) (for human participants or donors) - A statement that formal consent was obtained (must state whether verbal/written) OR the reason consent was not obtained (e.g., anonymity).**

>>> We have now included this in the method section of Study 1: *“Ethics Statement*

*The study was approved by University of Lausanne ethics committee (Project ID: C_SSP_082024_00012) and applies to the subsequent studies using the same data as well and complies with the Declaration of Helsinki for human subjects research. Electronic consent was obtained from all participants by ticking a box indicating their consent in the survey.”*

**3. Please upload separate figure files in .tif or .eps format. Also, remove the figures from your manuscript file but keep the legends.**

**For more information about figure files please see our guidelines:**

[**https://journals.plos.org/digitalhealth/s/figures**](https://journals.plos.org/digitalhealth/s/figures)

[**https://journals.plos.org/digitalhealth/s/figures#loc-file-requirements**](https://journals.plos.org/digitalhealth/s/figures#loc-file-requirements)

>>> We have done this.

**4. Please note that your Data Availability Statement is currently has a direct link to access each database but the link is directing us to the OSF homepage. If your manuscript is accepted for publication, you will be asked to provide these details on a very short timeline. We therefore suggest that you provide this information now, though we will not hold up the peer review process if you are unable.**

>>> We have made the project public now
